# Lifestyle and Quality of Life of Women with Diagnosed Hypothyroidism in the Context of Metabolic Disorders

**DOI:** 10.3390/metabo13101033

**Published:** 2023-09-25

**Authors:** Barbara Janota, Elżbieta Szczepańska, Kinga Noras, Ewa Janczewska

**Affiliations:** 1 Department of Basic Medical Sciences, Faculty of Public Health in Bytom, Medical University of Silesia in Katowice, 41-902 Bytom, Poland; 2 Department of Human Nutrition, Department of Dietetics, Faculty of Public Health in Bytom, Medical University of Silesia in Katowice, 41-808 Zabrze, Poland; 3 Department of Biometry, Warsaw University of Life Sciences, 02-787 Warsaw, Poland

**Keywords:** lifestyle, metabolism, hypothyroidism, quality of life, dietary habit, physical activity

## Abstract

The lifestyle causes of metabolic disorders in patients with hypothyroidism should be investigated. We aimed to assess the lifestyle and quality of life of women diagnosed with hypothyroidism and search for the presence of differences between the lifestyle and quality of life of women with and without diagnosed lipid metabolism disorders. This study included 311 women. To assess the differences between the groups with and without metabolic disorders, a non-parametric Mann–Whitney U test was performed. Of the products that were potentially beneficial for health, statistically significant differences in the average frequency of consumption were observed for legume seeds (*p* = 0.014), and of the products potentially unbeneficial for health, the frequencies of consumption of fried dishes (*p* = 0.016) and fast-food products (*p* = 0.001) were significant. Only 11.9% rated their free-time physical activity as high. The quality of life was significantly different between the groups. The lifestyle was moderately appropriate. Compared with women with lipid metabolism disorders, women without them exhibited a higher frequency of correct dietary behaviors regarding the consumption of products with a potentially beneficial effect and sleeping duration. Women without lipid metabolism disorders had a better quality of life. Women with hypothyroidism should be educated about the beneficial aspects of the regular consumption of vegetables, fruits, legumes, and fish and sleeping for the optimal amount of time.

## 1. Introduction

In hypothyroidism, thyroid gland function is affected by lifestyle, including eating behaviors and physical activity [1]. Notably, hypothyroidism predisposes patients to develop metabolic disorders, particularly lipid-related ones, which, however, develop only in specific types of patients [2,3]. Furthermore, thyroid hormones, as pleiotropic compounds, participate in the regulation of the distribution and metabolism of lipids in muscles, influencing the oxidation and transport of fatty acids in skeletal muscles [4]. The latest reports indicate the influence of thyroid hormones on the remodeling of the lipid profile, which influences muscle activity and endurance [5]. Therefore, the causes of metabolic disorders in patients with hypothyroidism should also be investigated in areas other than the thyroid gland, including lifestyle and physical activity, which may influence disease course and, consequently, affect quality of life [6,7,8]. This represents a modern, holistic research approach to the challenges individuals with hypothyroidism face. Moreover, it could facilitate an innovative approach to lipid metabolism disorders among at-risk groups.

Lifestyle behaviors are classified as potentially beneficial or potentially unbeneficial to health. Among the various behavioral factors, dietary habits seem to be the most important [9]. In the context of hypothyroidism, a diet should have anti-inflammatory and supportive effects on nutrient metabolism [10]. Therefore, vegetables and fruits should be consumed and food from natural sources and probiotics should be obtained [11,12,13,14,15,16]. To achieve this, vegetables and fruits should be consumed several times daily. Polyphenols, phytosterols, antioxidant vitamins, and trace elements in these food products exert anti-inflammatory effects and support the production of thyroid hormones [11,12,17]. Legumes and fish should be included in the diet several times a week as protein sources [13,14,15]. Fermented milk products are sources of probiotic bacteria and valuable proteins, and they should be consumed at least several times a week [16].

Food products that increase inflammation and disrupt lipid metabolism, including processed meat products having carcinogenic effects; fried products containing acrylamide (an increase in urinary concentration of acrylamide correlates with a decrease in thyroid hormone concentration in the blood); lard, which is a source of saturated fatty acids; sweet foods containing monosaccharides and trans fatty acids, which may increase the concentration of triglycerides and cholesterol; and fast food products [17,18,19,20,21,22] should be consumed in minimal quantities.

Other behaviors that affect thyroid function and metabolism include physical activity, sleep, and smoking [23,24,25,26,27]. Regular physical activity supports a reduction in body fat and the formation of muscle tissue, which is metabolically beneficial [28,29]. Sleep improves cell regeneration, and quitting smoking reduces oxidative stress and may improve mental well-being [25,26,30]. Concerns regarding mental health and related quality of life among chronically ill patients, including those with hypothyroidism, should be an inseparable element of therapy [31,32,33].

Owing to the relationship between hypothyroidism and lipid metabolism and the influence of lifestyle on the production of thyroid hormones, metabolism, and quality of life, the interdependencies discussed above must be analyzed. We aimed to assess the lifestyle and quality of life of women diagnosed with hypothyroidism and search for the presence of differences between the lifestyle and quality of life of women with and without diagnosed lipid metabolism disorders.

## 2. Materials and Methods

### 2.1. Characteristics of the Study Group

This study included 311 women diagnosed with hypothyroidism awaiting endocrinological consultation in Poland from November 2022 to January 2023. The inclusion criteria were female sex, ≥18 years of age, diagnosis of hypothyroidism, and provision of informed and voluntary consent for study participation. The exclusion criteria included age <18 years, pregnancy, diagnosis of other endocrinological disorders, diagnosis of diseases leading to wasting and requiring alternative or therapeutic diets, and the denial to provide informed and voluntary consent to participate. Data obtained from 310 women were analyzed. During the questionnaire verification process, one questionnaire was excluded due to incorrect completion. In the study group, 136 women (43.9%) reported the occurrence of lipid metabolism disorders (elevated levels of total cholesterol or triglycerides or the presence of fatty liver), whereas 174 women (56.1%) reported the absence of these disorders.

### 2.2. Research Tools

Standardized questionnaires supplemented with an author-created metric were used. They contained questions on age, height, body weight, waist circumference, hip circumference, education level, and coexisting diseases.

The KomPAN questionnaire, which was designed by the Committee on Human Nutrition Science of the Polish Academy of Sciences to study dietary views and habits, was used to assess the lifestyles of the study group participants [34]. Kowalkowska et al. reported on this tool’s repeatability in multicenter studies [35]. The questionnaire was used to assess dietary habits (number and regularity of meals, snack consumption, and sweetened beverage intake), frequency of consuming selected food products, and lifestyle factors (tobacco smoking, physical activity level, sleep time).

For assessing quality of life, questions from the WHOQOL-BREF questionnaire were selected. The WHOQOL-BREF questionnaire, created by the World Health Organization, includes questions related to psychological, somatic, environmental, and social domains [36].

### 2.3. Data Analysis

To assess differences in lifestyle and quality of life between the groups with and without metabolic disorders, a non-parametric Mann–Whitney U test was performed. The Spearman rank correlation coefficient was used to determine the correlation between dietary and non-dietary behaviors and the occurrence of disorders. For all analyses, a significance level of *p* < 0.05 was adopted. Microsoft 365 software, including R and Statistica13 programs for statistical analysis, was used to conduct the analysis.

### 2.4. Assessment of Diet Quality

Diet quality was determined according to the KomPAN questionnaire guidelines, which were adapted to the needs of the study. The Pro-Healthy Diet index was calculated by considering the frequency of consumption of the five selected food groups with potentially beneficial effects on health. These groups included fermented dairy beverages, fish, legume seeds, fruits, and vegetables.

Pro-Healthy Diet Index = (100/10) × sum of the frequency of consumption of the five food groups (times/day)

The Non-Healthy Diet Index was also calculated considering the five food groups with potentially detrimental effects on health. These groups included processed meat products, fried dishes, lards, fast foods, and sweet foods.

Non-Healthy Diet Index = (100/10) × sum of the frequency of consumption of the five food groups (times/day)

Responses regarding the frequency of consumption were assigned to appropriate categories, as presented in Table 1.

The method of interpretation of the obtained results for both indices is presented in Table 2.

Differences in the healthy and unhealthy diet indices between the groups were assessed using the Mann–Whitney U test.

## 3. Results

**Figure 1:**
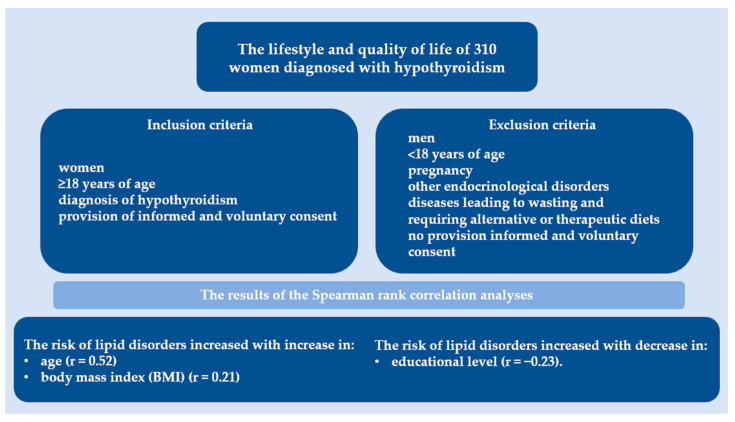
Overview of study design and key components. This figure summarizes the number of participants enrolled, outlines the inclusion and exclusion criteria, and presents the results of the correlation analyses.

Figure 1 summarizes this study, detailing the number of participants enrolled in the analysis, the inclusion and exclusion criteria, and the results of the correlation analyses.

### 3.1. Characteristics of the Study Group

Among the 310 women, 136 (43.9%) had lipid metabolism disorders. The participants were aged 18–86 years, and the mean age was 53 years. The educational levels of the participants were as follows: elementary education, 5.2%; vocational education, 17.1%; secondary education, 35.5%; and higher education, 41.9%.

Diabetes occurred in 16.2% and 3.4% of individuals with and without lipid metabolism disorders, respectively. Hashimoto’s disease accounted for 20.6% and 34.5% of individuals with and without lipid metabolism disorders, respectively. Statistically significant differences in the occurrence of both coexisting diseases were observed between the groups, with *p*-values of <0.001 and <0.007 for diabetes and Hashimoto’s disease, respectively.

Body mass index results are presented in Table 3.

A higher percentage of women without recognized lipid metabolism disorders (47.1%) had normal body weight than those with recognized disorders (30.9%). As per the Spearman rank correlation, the risk of lipid disorders increased with an increase in age (r = 0.52) and body mass index (BMI) (r = 0.21) and a decrease in educational level (r = −0.23).

### 3.2. Dietary Behaviors

The highest proportion of respondents consumed three or four meals per day (41.3% and 39.4%, respectively). A total of 41.6% reported eating meals regularly, and 8.7% did not consume snacks. Of all the respondents, 36.1% reported consuming sweetened drinks.

Vegetables were consumed at the recommended frequency of several times a day by 27.7% of the respondents, whereas fruits were consumed at least once a day by 36.8%. Plant legume seeds were consumed several times a week by 7.1% of the women, and fish was consumed at the same frequency by 12.6%. Fermented dairy products were consumed several times per week by 33.9% of the participants.

Of the product groups mentioned in Table 4, statistically significant differences in the average frequency of consumption were observed for legume seeds (*p* = 0.014).

Regarding waist and hip circumference, as the frequency of vegetable consumption increased, both waist and hip circumferences decreased (r = −0.18 and r = −0.13). Moreover, as the frequency of plant legume consumption increased, waist circumference decreased (r = −0.12). Furthermore, as the frequency of fish consumption increased, waist circumference decreased (r = −0.18).

Processed meat products and fried foods were consumed several times a week by 44.5% and 32.9% of respondents, respectively. Only 58.4% of women did not consume lard. Sweets were consumed at least several times a week by 53.6% of the participants, and fast food products were consumed at the same frequency by 1.6%.

Statistically significant differences in the average frequency of consumption of the product groups mentioned in Table 5 occurred in the frequency of consumption of fried dishes (*p* = 0.016) and fast-food products (*p* = 0.001).

As the frequency of processed meat product consumption increased, the waist and hip circumferences also increased (r = 0.18 and r = 0.21). As the frequency of lard consumption increased, hip circumference also increased (r = 0.13).

### 3.3. Assessment of Diet Quality

The average Pro-Healthy Diet Index scores were as follows: study group, 27.05; group with metabolic disorders, 25.7; and group without metabolic disorders, 28. The average Unhealthy Diet Index scores were as follows: study group, 16.1; group with metabolic disorders, 14.3; and group without disorders, 17.4. Overall, low levels of both pro-health and unhealthy dietary characteristics were observed. There were no statistically significant differences in the diet quality indices between the groups.

### 3.4. Non-Nutrition Behavior

In the study group, 81.3% of participants did not smoke. An optimal sleep duration of 6–9 h was achieved by approximately 64.2% and 68.4% of the respondents on weekdays and weekends, respectively. A total of 83.2% of women used electronic devices for less than 8 h daily, including working time. Only 11.9% of the respondents rated their free-time physical activity as high, as mentioned in Table 6.

Statistically significant differences were observed between groups in the number of hours of sleep per day on weekends (*p* = 0.001).

### 3.5. Quality of Life

Most women assessed their quality of life as good (59%). Approximately 39.7% were satisfied or very satisfied with their health status. In total, 63.6% of the participants were satisfied or very satisfied with themselves. With regard to intimate life, the highest percentage of women (40.6%) indicated that they were neither satisfied nor dissatisfied.

All the factors mentioned in Table 7 were significantly different between the groups (*p* = 0.037, *p* = 0.014, *p* = 0.037, and *p* = 0.002 for quality of life, health satisfaction, self-satisfaction, and intimate life satisfaction, respectively).

## 4. Discussion

Women diagnosed with hypothyroidism had shortcomings in their lifestyles that may adversely affect the function of the thyroid gland, predispose them to lipid metabolism disorders, and affect their quality of life. The lifestyles and qualities of life of the studied groups differed significantly. Women with hypothyroidism tended to be overweight. Thyroid hormone and TSH concentrations were positively correlated with BMI. Excessive BMI is associated with the accumulation of adipose tissue and the occurrence of lipid metabolism disorders [37,38,39]. In the study group, 58.4% of the women had BMIs above the normal range (50.5% without lipid metabolism disorders and 68.3% with lipid disorders). This highlights the need for weight and plasma lipid control, as well as need-based nutritional intervention among patients with hypothyroidism [40]. Ostrowska et al. reported that a low-calorie diet has a positive effect on the BMI and reduces body fat [41]. Considering the predisposition to metabolic disorders in women with hypothyroidism, the most frequently declared consumption (8.9%) of 3–4 meals a day and no snacks may be metabolically beneficial due to the imitation of intermittent fasting, which has a proven effect on lowering blood lipids [42].

The dietary behaviors of the study participants differed between the groups. The frequency of vegetable consumption was not sufficient in the overall population, and the recommended intake of several times a day was indicated by more women without lipid disorders than those with these disorders (31% and 23.5%, respectively). The same was true for the frequency of fruit consumption. In a cross-sectional analysis, Becerra-Tomás et al. reported from a study of 6633 people with metabolic syndrome that people who ate fruit more frequently were more likely to have a smaller waist circumference and lower low-density lipoprotein cholesterol levels [43]. The guidelines of the European Society of Cardiology (ESC) and European Atherosclerosis Society (EAS) for the management of dyslipidemia indicate the need to consume large portions of vegetables and fruits because of their anti-inflammatory and lipid-lowering effects [44].

Less than half of the respondents consumed fermented milk products at least several times per week. This frequency was 5.8% higher in women with lipid disorders than in those without these disorders. The consumption of dairy products at the same frequency was indicated by 69.2% of people in the study by Leonardo César de Freitas Cayres et al., who compared the eating behaviors of people with Hashimoto’s disease and healthy people to assess changes in the intestinal microbiome [45]. However, these differences may have resulted from differences in the populations’ eating styles (Poland vs. Brazil). Majid Ramezani et al., who studied the effect of synbiotics on the course of hypothyroidism, reported an improvement in the quality of life of patients despite the lack of effect of supplementation with a product containing post-biotic bacteria on the concentration of TSH in plasma. Therefore, the supply of probiotic bacteria through the consumption of fermented products is recommended [46].

The low frequency of consumption of fish by the study group, especially by women with lipid disorders, was found to be terrifying because fish are a source of iodine and selenium, which are particularly important for maintaining thyroid homeostasis [15]. Christine Tørris et al. indicated the beneficial effect of fish consumption on the lipid profile of women who ate fish more than once a week [47].

Our study investigated the frequency of consumption of processed goods, including processed meat products, fried products, and sweets. Zhang et al. studied the risk associated with the consumption of processed food among 8732 people and reported that the risk of subclinical hypothyroidism increased with the consumption of highly processed products [22]. Similar trends are expected in cases of overt hypofunctioning of this organ. Pagliai et al. examined the impact of processed food on health in a cross-sectional study and reported that the consumption of these foods was associated with a larger waist circumference, risk of excessive body weight, and lipid disorders [48]. Kaličanin et al., who studied the differences between the diets of people with Hashimoto’s hypothyroidism and those of healthy people, reported that those with the disease consumed more animal fats and processed meat [49].

The excessive consumption of monosaccharides in the study group may have contributed to the development of lipid metabolism disorders, including fatty liver, which can be counteracted by a diet low in monosaccharides, as shown by Schwimmer et al. in their experimental study. They reduced the sugar consumption of people with fatty liver, which resulted in a reduction in the accumulation of fatty compounds in the liver [50].

The non-nutritious lifestyle elements assessed in our study indicated an unsatisfactory level of physical activity and differences in sleep duration between the groups, indicating an insufficient number of hours of sleep, especially in women with lipid metabolism disorders. Appropriate sleep is crucial for proper metabolism. As indicated by Coppeta et al., who evaluated the impact of night work on thyroid function in their meta-analysis, night work may lead to an increase in TSH and predispose individuals to subclinical hypothyroidism [51]. An insufficient amount of sleep interferes with hunger and satiety centers, resulting in a decrease in the feeling of satiety, as shown by Ness et al. [52]. Moreover, Meguro et al., in their study of Japanese office workers, indicated that a shorter sleep duration was correlated with lower HDL cholesterol concentrations, which confirms its adverse effect on lipid metabolism [53]. Regarding physical activity, Sefat et al. examined the impact of physical activity on metabolic function among 20 girls with hypothyroidism and showed that the training group had a better body composition and BMI [54]. Babu et al. assessed the impact of physical activity on non-alcoholic fatty liver disease and showed a beneficial effect of exercise on the degree of steatosis, confirming the importance of physical activity in the occurrence of lipid metabolism disorders [55]. Werneck et al. reported the beneficial effect of aerobic physical activity on the quality of life of women with subclinical hypothyroidism [56].

Only 8.7% of women with hypothyroidism had a very good quality of life, including a higher percentage of women without lipid metabolism disorders. However, attention should be given to the multi-morbidity accompanying people with hypothyroidism, which most often results from chronic autoimmune inflammation of the thyroid gland [32]. Notably, in many papers, hypothyroidism itself is associated with dizziness, headache, depression, and arthritis, which can affect one’s quality of life [57,58,59,60]. Moreover, COVID-19 infection, which has affected many individuals, can result in metabolic disorders and thyroid dysfunction, including hypothyroidism and subclinical hypothyroidism, thereby exacerbating low quality of life [61].

Very high health satisfaction and intimate life satisfaction were also indicated by a higher percentage of women without lipid disorders. These results were confirmed by Hegedus et al., who described the impact of hypothyroidism on quality of life, indicating that the deteriorated quality of life of people with hypothyroidism may be caused by comorbidities [32,62]. The relatively low satisfaction with intimate life in women in our study may be explained by the possibility of sexual dysfunction among women with hypothyroidism [63,64].

## 5. Conclusions

The lifestyle of women with hypothyroidism was moderately appropriate. Compared with women with lipid metabolism disorders, women without them exhibited a higher frequency of correct behaviors regarding the consumption of products with a potentially beneficial effect on health and higher proportions of those sleeping for an appropriate number of hours a day and those engaging in physical activity. Women with lipid metabolism disorders exhibited a higher frequency of correct behaviors regarding the consumption of products that are potentially unbeneficial to health. Women without lipid metabolism disorders had a better quality of life.

Prevention programs for lipid metabolism disorders among women with hypothyroidism should focus on education about the beneficial aspects of the regular consumption of vegetables, fruits, legumes, and fish; undertaking physical activity; and sleeping for the optimal amount of time.

Strengths: This innovative and holistic approach to lipid metabolism disorders among at-risk groups presents areas in which lifestyle interventions could have a preventive effect.

Weaknesses: Owing to the limited number of publications in the analyzed area, our results were selectively compared with those of other studies. The consumptions of individual nutrients and their effects were not assessed using biochemical tests. However, we intend to do this in future studies.

Future directions for research should focus on conducting clinical and biochemical examinations among women and differentiate the types of metabolic disorders among the risk group.

**Figure 2:**
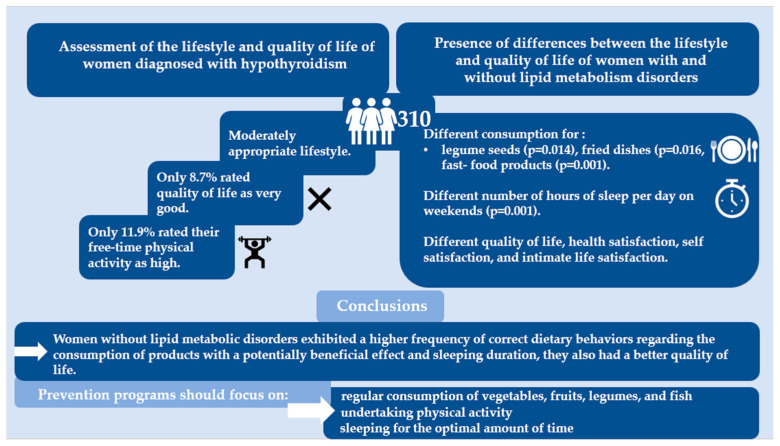
Graphical abstract.

Figure 2 shows the graphical abstract of the study.

## Figures and Tables

**Table 1:** Responses regarding the frequency of consumption assigned to appropriate categories.

Frequency of Consumption	Frequency (Times/Day)
Never	0
1–3 times a month	0.06
Once a week	0.14
Several times a week	0.5
Once a day	1
Several times a day	2

**Table 2:** The method of interpretation of the obtained results.

Intensity of Nutrition Features	Range (in Points)
Small	0–33
Moderate	34–66
High	67–100

**Table 3:** Body mass index.

BMI	Total *n* = 310		Group with Lipid Metabolism Disorders *n* = 136		Group without Lipid Metabolism Disorders *n* = 174		U Test *p*-Value
	*n*	%	*n*	%	*n*	%	
Underweight	5	1.61	1	0.7	4	2.3	0.001
Correct body weight	124	40	42	30.9	82	47.1	
Overweight	89	28.7	41	30.1	48	27.6	
Obese	92	29.7	52	38.2	40	23	

**Table 4:** Frequency of consumption of products potentially beneficial for health.

Product	Frequency of Consumption	Total *n* = 310		Group with Lipid Metabolism Disorders *n* = 136		Group without Lipid Metabolism Disorders *n* = 174	
		*n*	%	*n*	%	*n*	%
Vegetables	Never	2	0.6	1	0.7	1	0.6
	1–2 times a month	9	2.9	7	5.1	2	1.1
	Once a week	22	7.1	3	2.2	19	10.9
	A few times a week	90	29.0	36	26.5	54	31.0
	Once a day	100	32.3	57	41.9	43	24.7
	Several times a day	86	27.7	32	23.5	54	31.0
Fruit	Never	8	2.6	3	2.2	5	2.9
	1–2 times a month	14	4.5	6	4.4	8	4.6
	Once a week	31	10.0	7	5.1	24	13.8
	A few times a week	81	26.1	34	25.0	47	27.0
	Once a day	114	36.8	67	49.3	47	27.0
	Several times a day	61	19.7	19	14.0	42	24.1
Legumes	Never	53	17.1	33	24.3	20	11.5
	1–2 times a month	158	51.0	65	47.8	93	53.4
	Once a week	73	23.5	30	22.1	43	24.7
	A few times a week	22	7.1	7	5.1	15	8.6
	Once a day	3	1.0	1	0.7	2	1.1
	Several times a day	0	0.0	0	0.0	0	0.0
Fish	Never	23	7.4	10	7.4	13	7.5
	1–2 times a month	109	35.2	45	33.1	64	36.8
	Once a week	135	43.5	65	47.8	70	40.2
	A few times a week	39	12.6	14	10.3	25	14.4
	Once a day	3	1.0	2	1.5	1	0.6
	Several times a day	0	0.0	0	0.0	0	0.0
Fermented milk products	Never	35	11.3	16	11.8	19	10.9
	1–2 times a month	58	18.7	20	14.7	38	21.8
	Once a week	62	20.0	28	20.6	34	19.5
	A few times a week	105	33.9	50	36.8	55	31.6
	Once a day	48	15.5	21	15.4	27	15.5
	Several times a day	1	0.3	1	0.7	0	0.0

**Table 5:** Frequency of consumption of products potentially unbeneficial for health.

Product	Frequency of Consumption	Total *n* = 310		Group with Lipid Metabolism Disorders *n* = 136		Group without Lipid Metabolism Disorders *n* = 174	
		*n*	%	*n*	%	*n*	%
Processed meat	Never	20	6.5	11	8.1	9	5.2
	1–2 times a month	25	8.1	10	7.4	15	8.6
	Once a week	58	18.7	23	16.9	35	20.1
	A few times a week	138	44.5	51	37.5	87	50.0
	Once a day	54	17.4	34	25.0	20	11.5
	Several times a day	14	4.5	7	5.1	7	4.0
Lard	Never	181	58.4	85	62.5	96	55.2
	1–2 times a month	78	25.2	33	24.3	45	25.9
	Once a week	25	8.1	7	5.1	18	10.3
	A few times a week	15	4.8	6	4.4	9	5.2
	Once a day	7	2.3	2	1.5	5	2.9
	Several times a day	3	1.0	3	2.2	0	0.0
Fried foods	Never	28	9.0	14	10.3	14	8.0
	1–2 times a month	53	17.1	25	18.4	28	16.1
	Once a week	92	29.7	49	36.0	43	24.7
	A few times a week	102	32.9	38	27.9	64	36.8
	Once a day	32	10.3	10	7.4	22	12.6
	Several times a day	2	0.6	0	0.0	2	1.1
Sweets	Never	29	9.4	18	13.2	11	6.3
	1–2 times a month	56	18.1	27	19.9	29	16.7
	Once a week	58	18.7	24	17.6	34	19.5
	A few times a week	95	30.6	38	27.9	57	32.8
	Once a day	51	16.5	22	16.2	29	16.7
	Several times a day	20	6.5	7	5.1	13	7.5
Fast food	Never	136	43.9	82	60.3	54	31.0
	1–2 times a month	131	42.3	45	33.1	86	49.4
	Once a week	37	11.9	7	5.1	30	17.2
	A few times a week	3	1.0	0	0.0	3	1.7
	Once a day	2	0.6	2	1.5	0	0.0
	Several times a day	0	0.0	0	0	0	0.0

**Table 6:** Non-nutrition behaviors.

Chosen Behavior	Possible Answers	Total *n* = 310		Group with Lipid Metabolism Disorders *n* = 136		Group without Lipid Metabolism Disorders *n* = 174	
		*n*	%	*n*	%	*n*	%
Smoking	No	252	81.3	115	84.6	137	78.7
	Yes	57	18.4	21	15.4	36	20.7
Number of hours of sleep per day on weekdays	≤6	100	32.3	51	37.5	49	28.2
	>6–≤9	199	64.2	78	57.4	121	69.5
	≥ 9	10	3.2	7	5.1	3	1.7
Number of hours of sleep per day on weekends	≤6	77	24.8	48	35.3	29	16.7
	>6–≤9	212	68.4	80	58.8	132	75.9
	≥9	20	6.5	8	5.9	12	6.9
Number of hours spent using electronic devices (including work time)	<2	78	25.2	32	23.5	46	26.4
	2–<4	100	32.3	57	41.9	43	24.7
	4–<6	48	15.5	20	14.7	28	16.1
	6–<8	31	10.2	9	6.6	22	12.6
	8–<10	34	11.0	10	7.4	24	13.8
	≥10	18	5.8	8	5.9	10	5.7
Self-assessment of physical activity in free time	Low	126	40.6	58	42.6	68	39.1
	Moderate	146	47.1	59	43.4	87	50.0
	High	37	11.9	19	14.0	18	10.3

**Table 7:** Quality of life, health satisfaction, self-satisfaction, and intimate life satisfaction.

Chosen Element	Possible Answers	Total *n* = 310		Group with Lipid Metabolism Disorders *n* = 136		Group without Lipid Metabolism Disorders *n* = 174	
		*n*	%	*n*	%	*n*	%
Quality of life	Very bad	3	1.0	3	2.2	0	0.0
	Bad	6	1.9	3	2.2	3	1.7
	Neither good nor bad	90	29.0	45	33.1	45	25.9
	Good	183	59.0	76	55.9	107	61.5
	Very good	27	8.7	9	6.6	18	10.3
Health satisfaction	Very dissatisfied	12	3.9	7	5.1	5	2.9
	Not satisfied	59	19.0	31	22.8	28	16.1
	Neither satisfied nor dissatisfied	114	36.8	53	39.0	61	35.1
	Satisfied	115	37.1	43	31.6	72	41.4
	Very satisfied	8	2.6	2	1.5	6	3.4
Self-satisfaction	Very dissatisfied	12	3.9	5	3.7	7	4.0
	Not satisfied	25	8.1	9	6.6	16	9.2
	Neither satisfied nor dissatisfied	75	24.2	28	20.6	47	27.0
	Satisfied	141	45.5	63	46.3	78	44.8
	Very satisfied	56	18.1	31	22.8	25	14.4
Intimate life satisfaction	Very dissatisfied	16	5.2	7	5.1	9	5.2
	Not satisfied	22	7.1	11	8.1	11	6.3
	Neither satisfied nor dissatisfied	126	40.6	71	52.2	55	31.6
	Satisfied	87	28.1	28	20.6	59	33.9
	Very satisfied	56	18.1	19	14.0	37	21.3

## Data Availability

Data will be available by contacting the corresponding author. Data is not publicly available due to privacy.
